# Do Uniparental Sanderlings *Calidris alba* Increase Egg Heat Input to Compensate for Low Nest Attentiveness?

**DOI:** 10.1371/journal.pone.0016834

**Published:** 2011-02-09

**Authors:** Jeroen Reneerkens, Kirsten Grond, Hans Schekkerman, Ingrid Tulp, Theunis Piersma

**Affiliations:** 1 Centre for Ecological and Evolutionary Studies, Animal Ecology Group, University of Groningen, Groningen, The Netherlands; 2 SOVON, Dutch Centre for Field Ornithology, Nijmegen, The Netherlands; 3 Institute for Marine Resources and Ecosystem Studies, IJmuiden, The Netherlands; 4 Department of Marine Ecology, Royal Netherlands Institute for Sea Research, Texel, The Netherlands; Arizona State University, United States of America

## Abstract

Birds breeding in cold environments regularly have to interrupt incubation to forage, causing a trade-off between two mutually exclusive behaviours. Earlier studies showed that uniparental Arctic sandpipers overall spend less time incubating their eggs than biparental species, but interspecific differences in size and ecology were potential confounding factors. This study reports on a within-species comparison of breeding schedules and metal egg temperatures in uni- and biparental sanderlings (*Calidris alba*) in Northeast Greenland in relation to ambient temperature. We recorded incubation schedules with nest temperature loggers in 34 sanderling clutches (13 uniparentals, 21 biparentals). The temperature of a metal egg placed within the clutch of 17 incubating birds (6 uniparentals, 9 biparentals) was measured as an indicator of the heat put into eggs. Recess frequency, recess duration and total recess time were higher in uniparentals than in biparentals and positively correlated with ambient temperatures in uniparentals only. Uniparental sanderlings maintained significantly higher metal egg temperatures during incubation than biparentals (1.4°C difference on average). Our results suggest that uniparental sanderlings compensate for the lower nest attendance, which may prolong the duration of the incubation period and negatively affect the condition of the hatchlings, by maintaining a higher heat flux into the eggs.

## Introduction

Incubation is an energetically demanding phase of avian reproduction [1]–[3] and this is particularly so in High Arctic breeding birds of relatively small size that lay their eggs in open nest cups on the ground, often only a few centimeters above the permafrost [4]–[5]. Ambient temperatures in the High Arctic are usually far below the minimum of 26°C that is required for embryonic development [6], but when incubating adults forage they necessarily expose their clutch to ambient temperatures unless a partner takes over. This results in a conflict between the demands of incubating adults and those of their offspring [7]. The trade-off is especially relevant in uniparentally incubating adults that can not delegate incubation to a partner. As shorebirds are too small to have enough stores to rely on during incubation [8], they need to forage away from the nest to meet daily energy requirements [9]–[10]. When spending much time away from the nest, such birds run the risk of slowing down or even ceasing embryonic development [11].

Intermittent breeding schedules cause embryos to experience varying temperatures that negatively affect their growth rates, hatching condition and hatching success [2], [11]–[13] and lead to prolonged incubation periods with associated predation risk [14]–[17]. The incubating adult needs to put extra energy into reheating the eggs after a foraging bout [2]. Typically, partners that divide the incubation duties among themselves (hereafter: biparentals) experience a reduced cost of incubation [2] and their clutches will encounter less variation in egg temperature than clutches that are incubated by a single adult only (hereafter: uniparentals).

The rate of egg cooling will determine how long birds can leave their clutch unattended until the critical temperature is reached below which embryonic development will be impaired [6]. How fast eggs cool down depends, among other factors, on clutch size [13], [18], the temperature gradient between egg and the environment [10], [19], and the insulative properties of the lined nest cup [19]–[23]. All these factors may offer opportunities for the incubating bird(s) to limit the rate of egg cooling. Uniparental incubators might also be able to minimise the extension of the incubation period by maintaining a higher egg temperature during incubation bouts [16], [17]. In this study, we investigated two possible ways for uniparentals to enhance embryo development: (1) by adjustments of the incubation schedules in relation to ambient temperatures, and (2) by regulating egg temperature. Because the time that can be spent away from the nest will also depend on the rate with which food can be found, we also measured daily arthropod abundance.

**Figure 1:**
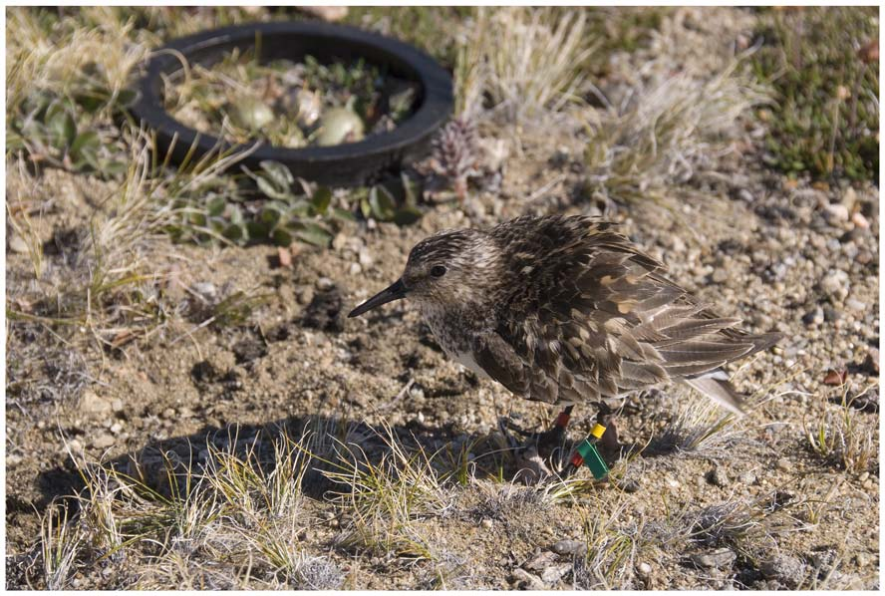
An individually color-ringed sanderling *Calidris alba* tries to lure away a researcher from its nest. Note the antenna loop around the clutch of four eggs in the background. The PIT is glued to the side of the extended green color-ring that is not visible on this photograph. Photo by Jeroen Reneerkens.

The consequences of, and adaptations to, uniparental incubation can best be studied in direct comparison to a biparental conspecific at the same location and time. The High Arctic breeding sanderling (*Calidris alba*; Fig. 1) is the only sandpiper known to exhibit both uni- and biparental incubation within the same breeding population [24], [25], but see [26]. Uniparental incubation in sanderlings is thought to be the result of mates of a pair dividing incubation between two clutches that are laid in rapid succession (‘double-clutching’) [27]. Because sanderlings have an almost invariable clutch size of four eggs [28], pairs that raise two clutches would thus have the potential advantage of a twice as large annual reproductive output. However, the associated uniparental incubation entails a higher energetic challenge due to constraints in time that can be spent off the nests [5] and/or may have consequences for the development and condition of the offspring due to the intermittent nature of incubation [11]. Given earlier between-species comparisons, we predicted that uniparental sanderlings would incubate their clutches less regularly. In this study, we compare the nest attendance of uni- and biparental sanderlings with those of other waders in Arctic, temperate and tropical climates. Because prolonged incubation duration entails certain fitness costs [14], [17], we also predicted that uniparentals compensate a lower nest attendance by an increase in egg temperature. A within-species comparison of breeding schedules and egg temperature of uni- and biparental parents might give insight into the evolutionary effects of different ecological conditions on breeding systems of sandpipers and avian reproduction in general.

## Methods

### Ethics Statement

The ethical guidelines promoted by the Association for the Study of Animal Behaviour were followed. The Greenland Ecosystem Monitoring Coordination Group at the National Environmental Research Institute, Aarhus University approved our detailed research proposals for our research activities in both 2007 and 2008. An exemption to the 'Executive Order no. 7 of 17 June 1992 from the Ministry of Domestic Affairs, Nature and Environment (Greenland Home Rule Authority) as amended by Executive Order no. 16 of 5 October 1999, to catch, (colour-)ring and take blood samples of birds and to travel into the National Park of North and Northeast Greenland in the summers 2007 and 2008 was obtained via Zackenberg Ecological Research Operations (ZERO) at the National Environmental Research Institute, Aarhus University.

### Study area and birds

Sanderling nests were studied between 6 June and 24 July 2007 and 30 May to 27 July 2008 in an area of ca. 24 km^2^ at Zackenberg, Northeast Greenland (74°30′ N 21°00′ W) [29]. Nests were located in a valley and on a south-facing mountain slope up to 550 m a.s.l. [30]. In both years 18% (six and seven in 2007 and 2008, respectively) of all clutches were still incomplete when we found them. Because all those clutches were preyed upon before they would have hatched, we were unable to determine incubation periods. The nest locations were registered by GPS and marked with a small plastic marker or stone pile ca. 10 m away from the nest. Hatching date was predicted by egg flotation (±2 days) [31] and start of incubation defined as 22 days before estimated hatch. We used an incubation length of 22 days based on common practice at Zackenberg. This value appeared to fit our observations well [32]. If an incomplete clutch was found, we considered incubation to start at clutch completion, which takes four consecutive days starting on the first egg date. The length and width of each egg in a clutch were measured with calipers to the nearest 0.1 mm. Egg volume was calculated using the formula length * width^2^ * 0.52 [33]. Clutch volume is the sum of egg volumes for each clutch.

Adult birds were caught on the nest with a small clapnet placed over the nest cup that was triggered by the returning bird. All caught birds were provided with a numbered metal ring on one of the tibia and plastic colored leg rings on the tarsi which made birds individually recognisable in the field. One of the color-rings was extended (as a ‘flag’). Blood samples were taken for molecular sexing [34].

### Identification of uni- or biparental clutches

**Figure 2:**
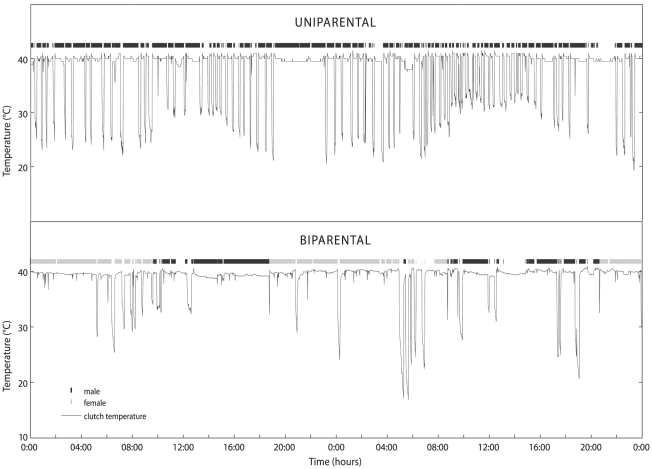
Two examples of an incubation pattern of 48 hours as registered by a temperature probe and by the PITs. Measured temperatures are depicted as a solid line, the presence of a PIT tagged male (black) and female (grey) as horizontal bars on top. The difference in incubation schedules with more recesses by the uniparental than the biparental clutch is clearly visible. Note that the PIT was not always detected when the temperature logger suggested an incubating bird (constant temperature around 40°C).

In 2007, we used transponders (PIT) in addition to thermologgers to help identify whether nests were uni- or biparental. A 10 mm long ×1.1 mm glass bead PIT was glued with plastic glue onto the extended color-ring of sanderlings. Twenty-six nests were fitted with PIT detecting antenna loops attached to a data logger to identify tagged individuals incubating them (Fig. 1). The loggers were programmed to register the presence and identity of a tagged sanderling on a nest every minute. Days during which nest visits by the researchers resulted in incubating sanderlings temporarily leaving the clutch were not included in the analyses. The antenna did not always detect an incubating bird; the thermologgers (see next subsection “Determination of incubation schedules”) regularly recorded periods with invariable temperatures around 40°C while at the same time no recordings were logged by the PIT loggers (Fig. 2). However, when the PITs were detected, the thermologgers always measured temperatures exceeding 36°C, so false positive identifications did not occur (Fig. 2). Because PITs were not always detected during incubation bouts, we only used the temperature recordings to determine incubation schedules. Because we had indications that mammalian predators easier found clutches incubated by PIT-tagged parents, we refrained from using them in 2008.

Clutches were considered to be incubated by two adult sanderlings (biparental) if two different birds (always a male and a female) were observed incubating those clutches. If only a single bird was observed at a nest, the PIT in combination with the thermologger could indicate the presence of an additional incubating bird if constant temperatures above 36°C were measured for several periods longer than an hour while the single bird with transponder was not detected. In 2008, when we did not use PIT's, we considered nine clutches uniparental, based on 4-8 nest visits (average 5.6) during which the same single incubating individual was encountered. On the biparental nests the second attending adult was usually encountered during the second visit to a nest (average visit no. 2.4, range 1–4).

Of the 68 sanderling nests found in 2007 and 2008, we obtained useful data on breeding schedules of 34 clutches. Most other clutches found either (1) fell victim to predation soon after discovery, (2) were found shortly before or at the day of hatch or (3) had the thermologger displaced. In 2007, 4 of 14 clutches, and in 2008, 9 out of 20 clutches were uniparental. Individuals are flexible in their strategy, as confirmed by three individuals that adopted different breeding strategies in different years (unpubl. data). However, we never obtained useful data of individual birds that were found incubating on two clutches within or between years. We monitored nest attendance for an average of 6±1.3 (SE) full days in uniparental clutches and 6.1±0.8 days in biparental clutches.

### Determination of incubation schedules

Incubation schedules were determined using small waterproof thermologgers (Tiny Tag, Gemini) that were placed near each nest and covered with soil and stones. A measuring probe (2×5 mm, temperature range −10°C to 50°C) was attached to the logger via thin electrical wire and fixed in the centre of the nest cup between the four eggs with a toothpick-sized piece of wood. The probe touched the brood patch of the incubating bird but we have no indication that it hampered its incubation. The loggers were programmed to take measurements every minute. After hatch, or when a clutch had been preyed upon, the loggers were collected and the data downloaded. Start and end of incubation bouts were determined by inspection of graphs of temperature against time (Fig. 2). Interpretation of the temperature graphs was straightforward because the relatively low ambient temperatures resulted in clear and immediate temperature drops when nests were left unattended (Fig. 2). As soon as temperatures were on the rise to levels higher than 36°C, incubation was presumed to have started. Occasional visual observations near nests of birds that started or ended an incubation bout always confirmed our interpretation of the temperature measurement. In three nests the probe became displaced and did not touch the bird's brood patch, resulting in temperature graphs that were difficult to interpret. These recordings were not included in our analysis.

### Heat input into eggs

A solid brass egg the size and shape of an average sanderling egg (35×25 mm) was placed in the nest cup and temporarily replaced one of the real eggs that was kept in a cup with cotton wool for the duration of the measurement. A probe, attached to a small thermologger (Tiny Tag), was mounted in the core of the metal egg. Metal egg temperature was recorded every 10 sec. Temperature was recorded for a period of at least 20 min continuous incubation which is sufficiently long to reach a stable temperature plateau due to the high heat conductance of brass.

**Figure 3:**
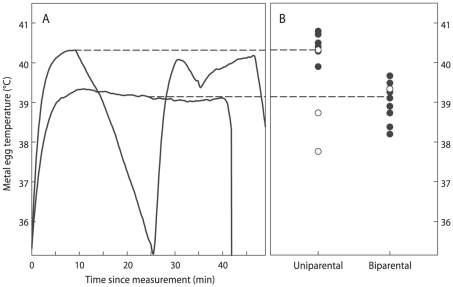
Metal egg temperature measurements in uni- and biparental clutches. Examples of temperature profiles during which a temperature plateau was or was not reached are shown in (A) and connected with a dashed line to the corresponding data point in (B). The final data are depicted in (B) where black solid circles indicate values of temperature profiles during which a plateau temperature was reached and the open circles where it was not.

We calculated metal egg plateau temperatures during incubation, by taking the average constant temperature. The period during which temperature was constant was defined to start when temperature did not increase for the first successive measurement. The end was the moment when the bird left the nest (Fig. 3). Four birds incubated for such short bouts that no stable temperature plateau was reached. Because of their higher recess frequency (see Results), this occurred more often in uniparentals (three times) than in biparentals (once). In those cases, the highest temperature recorded was used in the analyses and regarded as a conservative measurement (Fig. 3). We performed the statistical tests both with or without these measurements, but since this did not result in different qualitative outcomes of the statistical analysis, these measurements were included.

We aimed to measure metal egg temperatures on days 7 and 14 of incubation (if the start of incubation was not known it was estimated on the basis of egg flotation). If nests were discovered after day 14, measurements were made as soon as possible. Because nests were regularly discovered after day 7 of incubation or were preyed upon before day 14, we managed to measure metal egg temperature on both day 7 and 14 in only two clutches. We randomly selected only one brass egg temperature measurement for each of those two clutches and used those in further analyses. We used “metal egg temperature” as an indicator of heat put into the eggs by the incubating bird, which can be compared between individuals. We realize that a metal egg has a different heat conductance than a real sanderling egg and that metal egg temperature is also the result of heat loss to the environment. In our study we could not control heat loss from the metal egg to the environment, but a single metal egg and the same thermologger were used throughout the study.

### Ambient temperature

Air temperature was measured every five minutes by thermistors located at ground level at 20, 70, 150, 250 and 400 m altitude on the mountain slope where the sanderling nests were located. This slope was in the shade for several hours during the night resulting in lower night temperatures despite the continuous daylight. Temperatures did not differ significantly between the five locations (two-way ANOVA F = 0.003, df  = 4, P = 0.999) and we used the average temperature across all locations and averaged per hour in the analyses. Ambient temperatures varied between –1.7 and 28.5°C (average 12.7±6.5). Precipitation occurred rarely and is therefore not considered as an additional factor.

### Arthropod availability

In 2007 we set up five transects of eight-ten yellow plastic pitfall traps (diameter 10cm, depth 8 cm), each 1 m apart, to obtain a measurement of food availability for sanderlings. The transects were located at 20, 70, 150, 250 and 400 m altitude in habitat types in which sanderlings were seen foraging and close to where we found their nests. The traps were filled up with water to ca. 4 cm below the top. Half a teaspoon of salt was added to each trap to prevent freezing and decomposition of the arthropods. A drop of scentless detergent was added to each cup to break the surface tension. Between 19 June and 22 July 2007, arthropods were collected daily between 17:00 and 20:00 hrs and stored in 96% ethanol until analysis. Occasionally, some pitfall traps were destroyed by musk oxes *Ovibos moschatus*. Therefore, we express insect abundance per pitfall per day for all transects combined. Invertebrates were identified up to order, and often also family level, and measured to the nearest mm. Dry weights were estimated from group specific length- dry weight relationships that were established based on subsamples of the collected arthropods in 2007. Mites (Acari) and springtails (Collembola) were considered too small to be important as prey for sanderlings and were not taken into account in the analysis.

### Statistical analyses

To account for the hierarchical structure of our data (e.g. multiple hourly observations per day, multiple days per nest), analyses were performed using the multivariate multilevel mixed-modeling procedure in MLwiN 2.15 [35]. This method decomposes the total variance of the dependent variables into three levels (between nests, days and observations) by including ‘nest’ and ‘day’ as random effects (intercepts). The model was used to estimate the effects of ambient temperature and ambient temperature squared (continuous fixed effects) on incubation schedules, but we did not account for the random variation in slope and intercept of temperature on the dependent variables. The fixed effects were modelled as separate coefficients. Ambient temperature squared was included in the model because we expected the three variables to rise until a peak at high ambient temperatures, above which they would decrease again due to a decreased energy demand of parents. Incubation stage was also included as fixed effect (both linearly and the second order polynomial), but was eliminated from the final model because it did not have significant effects. Incubation schedules were described by the dependent variables ‘total recess time’ (in minutes per hour), ‘recess frequency’ (number of recesses per hour) and average recess length (minutes per hour) for both uni- or biparental clutches. We used periods of an hour to study the effects of ambient temperature on incubation behaviour within days. Earlier observations had shown that sanderlings regularly leave their clutches within periods of an hour.

The effect of ambient temperature on total recess time, recess frequency and average recess length was investigated at the level of clutch, per day nested within clutch and per hour nested within clutch and day. All explanatory variables were centred on their averages. To meet normality assumptions, recess time was arcsine transformed and we took the natural logarithms ln (x+1) of the number of recesses hour^−1^. All two-way interaction terms were included in the model initially and then all insignificant interaction terms were removed sequentially (backward deletion) by decreasing P value. The removal of interactions did not change the significance of main effects.

We used an analysis of covariance to test for the effects of day of incubation, current ambient temperature, clutch volume and number of incubating adults on metal egg temperature. All two-way interactions were initially included in the model. Non-significant variables were removed from the model, starting with the interaction terms. All tests were two-tailed and threshold values of P = 0.05 were applied throughout.

## Results

### Incubation schedules

**Figure 4:**
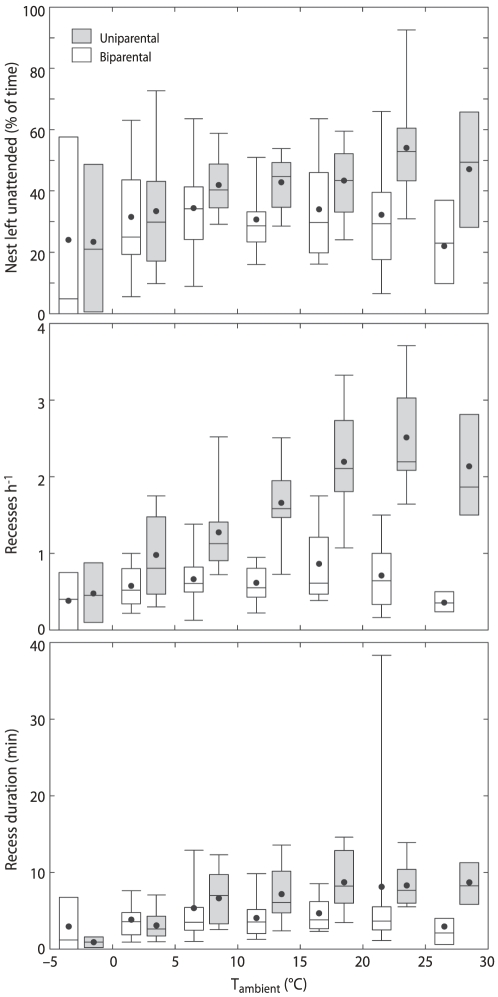
Average percentage of time that clutches were left unattended, recess frequency and recess duration of uniparentals and biparentals. Uniparentals are indicated by grey boxes, biparentals by white boxes. For presentation purposes the hourly ambient temperatures are catagorized per 5°C. The graphs are based on the raw data. The dots indicate the average, the boxes encloses 50% of the data and the error bars 95%.

Both uni- and biparentals regularly left their clutches unattended (Figs 2 and 4). When we observed color-ringed sanderlings during an off-bout they usually were foraging (cf. [10]), but we ocassionally also observed sanderling males leaving their nest to chase away other sanderlings in the vicinity, as was also observed by [36]. Uniparentals spent more time off their nest (42.1±6.3%) than biparentals (32.2±14.5%) and this difference almost reached 5%-statistical significance (Table 1). The average for biparentals is influenced by a single unusual clutch that was left unattended for 68% of the 72 hours of available data. Excluding this value makes the time spent off the nest by biparentals 30±11.3%, and the difference with uniparentals significant (Chi-square  = 3.94, df = 1, P = 0.047). The difference was brought about by the more frequent recesses as well as longer recess durations of uniparental sanderlings compared to biparental sanderlings (Table 1, see Fig. 2 for an example).

**Table 1:** Multivariate model summaries examining total recess time (minutes hour^−1^), recess frequency (recesses hour^−1^) and recess duration for uniparental relative to biparental (reference category) sanderling clutches.

Response variable					
	Fixed effect	β	(SE)	χ^2^	P
Total recess time					
	Intercept			**36.1**	**<0.001**
	Breeding strategy	8.34	(4.51)	3.43	0.06
	T_ambient_ (hour)	0.20	(0.19)	1.13	0.29
	T_ambient_^2^ (hour)	−0.005	(0.007)	0.51	0.47
					
	Breeding strategy * T_ambient_ (hour)	**2.13**	**(0.29)**	**53.6**	**<0.001**
	Breeding strategy * T_ambient_^2^ (hour)	**−0.02**	**(0.01)**	**4.73**	**0.03**
Recess frequency					
	Intercept			**141.2**	**<0.001**
	Breeding strategy	**0.43**	**(0.05)**	**64.2**	**<0.001**
	T_ambient_ (hour)	**0.02**	**(0.004)**	**29.2**	**<0.001**
	T_ambient_ (day)	0.00	(0.004)	0.00	1.00
	T_ambient_^2^ (hour)	**−0.001**	**(0.00)**	**32.1**	**<0.001**
	Breeding strategy * T_ambient_ (day)	**0.02**	**(0.005)**	**19.9**	**<0.001**
	Breeding strategy * T_ambient_ (hour)	**0.04**	**(0.002)**	**261.3**	**<0.001**
Recess duration					
	Intercept			**58.5**	**<0.001**
	Breeding strategy	**3.01**	**(0.91)**	**10.9**	**0.001**
	T_ambient_ (nest)	**−1.31**	**(0.45)**	**8.69**	**0.003**
	T_ambient_ (hour)	0.05	(0.06)	0.50	0.48
	T_ambient_^2^ (nest)	**0.05**	**(0.02)**	**6.08**	**0.01**
	Breeding strategy * T_ambient_ (hour)	**0.30**	**(0.10)**	**8.66**	**0.003**

Significant factors are in bold. The final model after elimination of the non-significant interaction terms is shown, but note that non-significant main effects were kept in the model if their interaction terms were significant.

Ambient temperature at the level of hour positively affected recess frequency but did not have an overall effect on total recess time and recess duration. The significant interactions between breeding strategy and ambient temperature at the level of hour indicates that breeding schedules of uniparentals were affected by ambient temperatures, whereas they were not in biparentals (Table 1, Fig. 4). At lower temperatures all three descriptors of incubation patterns were similar between uni- and biparentals, but with increasing temperatures uniparentals increased recess frequency and recess duration (Fig. 4). This resulted in longer periods during which uniparentals left their clutch unattended at higher ambient temperatures (Table 1, Fig. 4). The interaction between breeding strategy and ambient temperature was also significant on the level of day for recess frequency, but not for recess duration and total recess time (Table 1). Recess frequency per hour leveled off and slightly decreased with higher ambient temperatures (Fig. 4), indicated also by the significant effect of squared ambient temperature at the level of hour (Table 1).

### Metal egg temperature

Day of incubation (Ancova, F_15,1_  = 0.323, P = 0.58), clutch volume (F_16,1_ = 0.495, P = 0.49) and ambient temperature (F_17,1_ = 1.098, P = 0.31) did not effect metal egg temperatures, but whether clutches were incubated by one or two parents did (F_17,1_ = 6.563, P = 0.02). Metal egg temperatures were on average 0.8°C higher in uni- compared to biparentals (Fig. 3). If the analysis is restricted to measurements in which a temperature plateau was reached only, the average difference is more pronounced (1.4°C; F_13,1_ = 33.55, P<0.001).

## Discussion

Our data confirm earlier observations [30] that clutches of sanderlings in northeast Greenland are incubated either by one or by two parents, although uniparental clutches were less common (13 out of 34 nests). Recess time in the uniparental sanderlings (42.1%) was considerably higher compared to what was found in other uniparental shorebirds in arctic or temperate regions (Table 2). Also the recess time of 32.2% in biparental sanderlings exceeded the range of other arctic and temperate biparental shorebirds, but fell within the range of tropical biparental shorebirds (Table 2). Recess time can be affected by several factors, such as energetic requirements of birds, food availability, insulation of the nest cup and climatic conditions [10], [37]. Compared with many other High Arctic regions, in northeast Greenland ambient temperature is relatively high and air humidity is low [38], leading to a relatively slow heat loss of eggs, which may explain why the frequent and long recesses of sanderlings recorded in our study were possible.

**Table 2:** Average nest attentiveness, recess frequency and recess duration per 24 hrs of uni- and biparentally incubating shorebirds in different climates.

Breeding strategy	Climate	Species	Nest attentiveness	Recess frequency	Recess duration (min)	Method	Ref
Uni	Arc	*Calidris ferruginea*	0.82±0.07	21.6±7.4	15.3±19.5	T_nest_	[10]
Uni	Arc	*Calidris fuscicollis*	0.83	25.1	10.5	camera	[9]
Uni	Arc	*Calidris melanotus*	0.82±0.01	28.8±1.4	6.0	T_nest_	[20]
Uni	Arc	*Calidris melanotus*	0.85±0.01			T_nest_/T_egg_	[50]
Uni	Arc	*Calidris melanotus*	0.83±0.06	29.9±9.2	9.8±9.0	T_nest_	[10]
Uni	Arc	*Calidris minuta*	0.81±0.08	26.5±4.4	9.4±8.6	T_nest_	[10]
Uni	Arc	*Charadrius morinellus*	0.90±0.03			T_nest_	[52]
Uni	Arc	*Charadrius morinellus*	0.83			T_nest_	[53]
Uni	Temp	*Gallinago media*	0.90±0.02	15.7±6.1	8.7±1.9	T_nest_	[54]
Uni	Arc	*Phalaropus fulicarius*	0.87±0.02		7.2±1.0	T_nest_	[10]
Uni	Arc	*Phalaropus fulicarius*	0.81		6.6	camera	[55]
Uni	Temp	*Rostrathula bengalensis*	0.84			obs	[56]
**Range (excluding this study)**			**0.81–0.90**	**15.7–29.9**	**6.0–15.3**		
**Uni**	**Arc**	***Calidris alba***	**0.57±0.1**	**64.6±14.1**	**7.2±2.1**	**PIT/T_nest_**	**This study**
Bi	Arc	*Calidris alpina*	0.98±0.00		6.0±1.0	T_nest_/T_egg_	[51]
Bi	Arc	*Calidris bairdii*	0.97±0.00		6.0±1.0	T_nest_/T_egg_	[51]
Bi	Arc	*Calidris pusilla*	1.00±0.00		1.5±0.5	PIT/T_nest_	[22]
Bi	Arc	*Charadrius morinellus*	0.91±0.03			T_nest_	[52]
Bi	Arc	*Charadrius morinellus*	0.96			obs	[53]
Bi*	Temp	*Vanellus vanellus*	0.84±0.02			camera	[57]
**Range (excluding this study)**			**0.84–0.98**		**1.5–6.0**		
**Bi**	**Arc**	***Calidris alba***	**0.71±0.1**	**38.7±7.2**	**5.1±3.8**	**PIT/T_nest_**	**This study**
Bi	Trop	*Charadrius alexandrinus*	0.86±0.01			obs	[58]
Bi	Trop	*Charadrius peronii*	0.67			obs	[59]
Bi	Trop	*Charadrius wilsonia*	0.83			obs	[60]
Bi	Trop	*Vanellus coronatus*	0.99			T_nest_/obs	[61]
Bi	Trop	*Vanellus lugubris*	0.91			T_nest_/obs	[61]
Bi	Trop	*Vanellus melanopterus*	0.89			T_nest_/obs	[61]
**Range (excluding this study)**			**0.67–0.99**				

Climate is subdivided into arctic (‘Arc’), temperate (‘Temp’) or tropical (Trop'). In bold are the ranges of average values for uniparentals and biparentals in Arctic or temperate regions and for biparentals in tropical regions summarized. They are compared with the values for sanderling based on this study (also in bold). Abbreviations for recording methods refer to visual observation (obs), camera surveillance (camera), recording of nest (T_nest_) or egg temperature (T_egg_), or recording of presence of PIT-tagged parents (PIT).

*Based on 20 clutches of which two were never attended by a male and were thus uniparental, and 10 others only attended by males during daytime.

Despite the relatively high overall recess time in sanderlings, there was a clear difference between uni- and biparental clutches in recess time and the effect of ambient temperature on breeding schedules. This reconfirms that the trade-off between incubation and foraging is more severe for uniparentals because incubation duties cannot be taken over by a partner while foraging. By foraging during frequent but short bouts, and by timing such recesses during the warmer periods of the day, uniparentals can probably minimize the variation in egg temperatures, which increases energy use of the embryo and negatively affect hatchling phenotype [11]. Experimental heating of nests resulted in higher nest attendance in biparental sandpipers [20] and uniparental tree swallows *Tachycineta bicolor* [39]. This suggests that incubation schedules are determined by the energetic trade-offs of the incubating birds rather than optimization of the conditions for embryonic development. Our correlative data suggest a different response to higher temperature, but this in part reflects the within-day organization of foraging recesses, concentrated in the warmer mid-day hours.

Leaving the clutch unattended during the warmest periods of the day is probably intended to keep suboptimal conditions for embryonic growth to a limit, but also suggests that the possibility for sanderlings to raise a clutch alone depends to a certain degree on environmental circumstances and the condition of the incubating bird. Indeed, three uniparental, but no biparental, sanderlings were observed to abandon their nests before hatch during severe weather conditions with low ambient temperatures, high wind speed and snowfall (JR unpubl. obs. and see [26]). In a different study, uniparental sandpipers were shown to considerably decrease in body mass during periods with low temperatures, while a biparental species did not [10].

**Figure 5:**
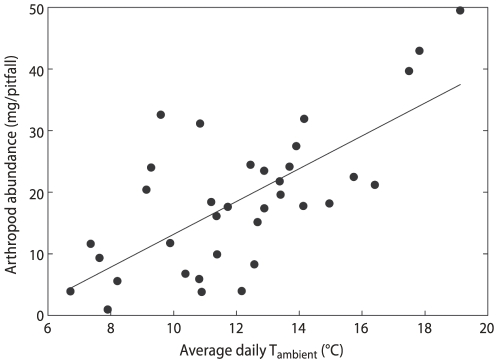
Relationship between daily mean ambient temperature and the average dry weight of arthropods collected per pitfall trap. The linear regression is statistically significant (F_1_ = 31.2, P<0.0001, R^2^ = 0.486).

In addition to the need to provide a favourable physical environment for the developing embryo, foraging success may also determine incubation schedules, especially in uniparental sanderlings. Arthropod availability on the Arctic tundra is strongly influenced by daily ambient temperature [40], [41] and this was confirmed in our study (Fig. 5). This indicates that foraging during the warmer periods of the day has both the advantage of a slower cooling of the clutch and potential higher food intake rate by the foraging adults. That uniparental sanderlings increase recess frequency, recess duration and total recess time with ambient temperatures at the level of hour, but not consistently for the three variables at the levels of day and nest (Table 1), may indicate that the birds need to forage daily for a minimum period of time regardless of the daily average temperature, but that if there is the possibility, the warmest period within days is chosen to leave the nest. An alternative, but not mutually exclusive, explanation is that the birds compensate for daily fluctuations in their energy balance with stored energy to avoid too much variation in incubation constancy between days [10].

Adjusting breeding schedules to ambient temperatures probably helps uniparental sanderlings minimize fluctuations in egg temperatures while still meeting energetic demands. However, overall nest attendence of uniparental sanderlings was 9.9% lower compared to biparentals. Leaving a clutch unattended slows down embryonic growth and therefore lengthens the incubation period [6], [12], [15], [42]. Starling *Sturnus vulgaris* clutches that were incubated by both parents, and hence received a higher attendance, indeed had shorter total incubation periods [2]. A longer incubation period has negative implications for current and future reproductive success arising from higher energetic costs for the parents and an increased probability of clutch predation [2], [17], [43], [44]. The latter is a serious factor, as predation rates on shorebird nest can be considerable in the High Arctic [45], [46]. A longer incubation period may also have negative consequences for the offspring, through a poorer condition at hatch due to greater energy expenses during the longer incubation period [11], [42], [47], [48].

We argue that the higher heat input into the eggs by uniparental than biparental sanderlings, indicated by the higher measured metal egg temperatures, could be a way to compensate for the effect of lower incubation constancy on incubation duration, but future experiments are needed to confirm our correlative data. By maintaining higher incubation temperatures, parents can efficiently shorten incubation duration and positively modify offspring phenotype [16], [39], [49]. Maintaining high egg temperatures is likely to be energetically costly and can probably only be performed by experienced individuals in good physical condition, as suggested by a study on biparental king penguins *Aptenodytes patagonicus* that were experimentally prevented from sharing incubation [50]. In the absence of a helping partner, birds incubated longer than control birds and kept egg temperature constant, but egg temperature significantly decreased two days before it was finally abandoned.

We have demonstrated that uniparental birds keep a tighter breeding schedule than biparentals and, for the first time under natural conditions, that they put more body heat into their clutches. These possible adaptations must come with some costs, raising the question of why some sanderlings incubate clutches without the help of a partner. Double-clutching would have the benefits of a possibly twice as large reproductive success in a given breeding season. In the relatively benign climate in northeast Greenland, uniparental incubation is apparently feasible, but possibly only by high quality individuals. The existence of this double-clutching breeding system has, however, never been convincingly proven [25] and if it would occur, it is possible that males and females profit unequally if extra-pair paternity occurs too. Molecular parentage analysis is needed to gain more insight into the breeding system of sanderlings and to evaluate the costs and benefits of uniparental incubation under different environmental conditions.
